# Metabolic syndrome incidence in an aging workforce: Occupational differences and the role of health behaviors

**DOI:** 10.1016/j.ssmph.2021.100881

**Published:** 2021-07-28

**Authors:** Katharina Runge, Sander K.R. van Zon, Ute Bültmann, Kène Henkens

**Affiliations:** a Netherlands Interdisciplinary Demographic Institute, Lange Houtstraat 19, 2511 CV, The Hague, the Netherlands; b Department of Health Sciences, Community and Occupational Medicine, University of Groningen, University Medical Center Groningen, Hanzeplein 1, 9700 RB Groningen, the Netherlands; c Faculty of Social and Behavioral Sciences, University of Amsterdam, Nieuwe Achtergracht 129-B, 1018 WT Amsterdam, the Netherlands

**Keywords:** Older workers, Endocrinology, Occupation, Lifestyle factors

## Abstract

This study investigates whether the incidence of metabolic syndrome (MetS), and its components, differs by occupational group among older workers (45–65 years) and whether health behaviors (smoking, leisure-time physical activity, diet quality, and alcohol consumption) can explain these differences. A sample of older workers (N = 34,834) from the North of the Netherlands was investigated. We analyzed data from two comprehensive measurement waves of the Lifelines Cohort Study and Biobank. MetS components were determined by physical measurements, blood markers, medication use, and self-reports. Occupational group and health behaviors were assessed by questionnaires. The association between occupational groups and MetS incidence was examined using logistic regression analysis. Health behaviors were subsequently added to the model to examine whether they can explain differences in MetS incidence between occupational groups. Low skilled white-collar (OR: 1.24; 95 % CI: 1.12, 1.37) and low skilled blue-collar (OR: 1.37; 95 % CI: 1.18, 1.59) workers had a significantly higher MetS incidence risk than high skilled white-collar workers. Similar occupational differences were observed on MetS component level. Combinations of unhealthy behaviors were more prevalent among blue-collar workers. MetS incidence in older workers differs between occupational groups and health behaviors explain a substantial part of these differences. Health promotion tailored to occupational groups may be beneficial specifically among older low skilled blue-collar workers. Research into other factors that contribute to occupational differences is needed as well as studies spanning the entire working life course.

## Highlights

• Occupational group membership is associated with MetS incidence among older workers.
• Low skilled blue-collar workers have the highest MetS incidence risk.
• Findings are similar for the individual MetS components.
• Unhealthy behaviors cluster most among blue-collar workers.
• Health behaviors explain a large part of the occupational MetS risk differences.

## 1 Introduction

The metabolic syndrome (MetS) is a major public health concern (Eckel, Grundy, & Zimmet, 2005; Saklayen, 2018). MetS is a cluster of at least three of the following risk factors: hypertension, abdominal obesity, raised fasting plasma glucose, raised triglycerides, and lowered high-density lipoprotein (HDL) cholesterol (Alberti et al., 2009). Already in 2005, MetS was exclaimed an emerging global epidemic with prevalences ranging from 5.6 % up to 67 % depending on the ethnic or age group under consideration (Eckel et al., 2005). In 2018, the estimated global prevalence of MetS was 25 % with an increasing trend (Saklayen, 2018). MetS incidence resembles Type 2 diabetes mellitus (T2DM) incidence, which increases with age and peaks at 25 % among the age of 65 years and older (Saklayen, 2018). Having MetS is associated with a two-fold risk of developing cardiovascular disease (CVD), a five-fold risk for T2DM (Alberti et al., 2009; Lee et al., 2020), and increased mortality (Mottillo et al., 2010). Although MetS encompasses a combination of at least three risk factors, the major driving force is the MetS component abdominal obesity, related to the global obesity epidemic (Eckel et al., 2005; Wang & Beydoun, 2007). Additionally, insulin resistance is involved in the pathophysiology of MetS, which is facilitated by adipose tissue and further related to the MetS component hypertension (Eckel et al., 2005). The best intervention route seems to be weight reduction as this improves all MetS components and reduces the risk of T2DM (Eckel et al., 2005). Consequently, when studying MetS, it is important to investigate not only the overall syndrome but also the individual underlying components.

Older workers might be especially at risk for MetS incidence for at least three reasons. First, MetS risk increases with age (Sánchez-Chaparro et al., 2008) and there is a linear age increase in the central MetS component abdominal obesity (Wang & Beydoun, 2007). Second, statutory retirement ages are increasing and people stay in the workforce until an older age (Soltysik, Kostka, Karolczak, Watala, & Kostka, 2019). Third, the effect of unhealthy behaviors like smoking, physical inactivity, an unhealthy diet, and high alcohol consumption, which are assumed to play a causal role in MetS incidence (Eckel et al., 2005; Pérez-Martínez et al., 2017), might have accumulated over the life-course and could lead to a stronger detrimental health effect among older workers (Singh-Manoux, Ferrie, Chandola, & Marmot, 2004).

It is important to investigate MetS and the individual MetS components among older workers as MetS related consequences like CVD and T2DM are related to an increased risk for early work exit and disability benefits (Kouwenhoven-Pasmooij, Burdorf, Roos-Hesselink, Hunink, & Robroek, 2016). Further, the number of MetS components at the age of 60 is significantly associated with early death after retirement (Sakurai et al., 2020) and the quality of life in retirement can be affected by CVD and T2DM (Laiteerapong et al., 2011; Schofield et al., 2012). A recent literature review reported that most studies about MetS among workers are cross-sectional and focus on the general working population (Santana, das Merces, Magalhães, Costa, & D’Oliveira, 2020).

Cross-sectional research shows that MetS prevalence rates are differentially distributed among white- and blue-collar occupations. White-collar workers generally perform office work, examples are service workers or managers, whereas blue-collar workers usually work in a non-office environment, like machine operators or trade workers (Brønnum-Hansen, Foverskov, & Andersen, 2020; Gilson et al., 2019). MetS rates are generally higher among socio-economically disadvantaged groups like low skilled and blue-collar workers Davila et al., 2010, Hidaka et al., 2016, Robinson et al., 2020, Sánchez-Chaparro et al., 2008, Santana, das Merces, Magalhães, Costa, & D’Oliveira, 2020, Soltysik et al., 2019. The same pattern has been observed on MetS component level (Davila et al., 2012, Proper and Hildebrandt, 2010). For instance, obesity rates are higher among trade and transportation workers (Proper & Hildebrandt, 2010) and hypertension is more common among protective service workers (Davila et al., 2012). However, cross-sectional studies do not allow for conclusions about whether an occupation, or an occupational group, influences MetS incidence or if MetS differences are due to a selection of unhealthy individuals into certain jobs. It is necessary to investigate MetS longitudinally to identify risk factors for MetS incidence. A recent longitudinal study among workers of all ages showed that occupational group membership is not only related to MetS prevalence but also to MetS incidence with the highest associated MetS risk among blue-collar workers (van Zon, Amick III, de Jong, Brouwer, & Bültmann, 2020).

Differences in the distribution of MetS and its components across occupational groups might be explained by a higher prevalence, and clustering, of unhealthy behaviors in specific occupational groups (Thebault et al., 2018; Väisänen et al., 2020). Further, exposure to, and possible accumulation of, unhealthy behaviors over the life-course (Lynch, Kaplan, & Salonen, 1997; Singh-Manoux et al., 2004) could have a profound detrimental health effect among older workers. Key health behaviors related to MetS and its components are smoking, physical activity, diet, and alcohol consumption (Pérez-Martínez et al., 2017). Smoking is associated with abdominal obesity, reduced HDL-cholesterol, and increased triglycerides resulting from higher levels of inflammatory biomarkers (Pérez-Martínez et al., 2017). Physical inactivity is related to abdominal obesity, hypertension, raised fasting glucose, triglycerides, and inflammation. An unhealthy diet is associated with all MetS components (Pérez-Martínez et al., 2017). Lastly, a moderate intake of red wine and beer is related to a lower overall MetS prevalence due to the favorable effect on abdominal obesity, reduced HDL-cholesterol, hypertension, and raised fasting glucose (Pérez-Martínez et al., 2017). However, this favorable effect is reversed for high alcohol consumption and for other alcohol types like liquors or spirits (Pérez-Martínez et al., 2017). Higher rates of unhealthy behaviors are observed among low skilled workers and blue-collar occupations than among high skilled workers and white-collar occupations (Gilson et al., 2019; Howard, 2004; Lynch et al., 1997; Thebault et al., 2018; Turrell, Hewitt, Patterson, Oldenburg, & Gould, 2002; Väisänen et al., 2020).

The current study aims to contribute to the literature in at least three ways. First, we will examine differences in the incidence of MetS and its components in older workers. This adds to the gap of longitudinal findings among the aging workforce (Santana et al., 2020). Second, we will examine to what extent differences in MetS incidence can be explained by health behaviors. Additionally, combinations of unhealthy behaviors across occupational groups, and the association of combinations of unhealthy behaviors with MetS incidence, will be examined as poor health behaviors tend to cluster. This may offer employees, employers, and occupational healthcare workers with potential levers for preventive measures. Third, we will longitudinally analyze large-scale panel data of the Lifelines Cohort Study and Biobank (N = 34,834). Lifelines offers the unique opportunity to combine objectively measured health outcomes with extensive questionnaire data (Scholtens et al., 2015).

## 2 Methods

### 2.1 Study design and sample

The current study was embedded within the Lifelines Cohort Study and Biobank (Scholtens et al., 2015). Lifelines is a multi-disciplinary prospective population-based cohort study examining the health and health-related behaviors of 167,729 persons living in the North of the Netherlands. It employs a broad range of investigative procedures in assessing the biomedical, socio-demographic, behavioral, physical, and psychological factors which contribute to the health and disease of the general population, with a special focus on multi-morbidity and complex genetics. The ongoing data collection started with a comprehensive baseline assessment at one of the Lifelines research centers, with the completion of questionnaires, the collection of biological samples, and a physical examination (Scholtens et al., 2015). The second comprehensive assessment took place after approximately 5 years (Scholtens et al., 2015).

Data were used from adult participants at the comprehensive baseline assessment (N = 152,728) and their follow-up data from the second comprehensive assessment. Inclusion criteria were: older workers (i.e. 45–65 years old at baseline (WHO Study Group on Aging & Working Capacity, 1993), working at least 12 h per week (Janssen & Dirven, 2015), no drop-out between baseline and follow-up assessment, and no missing data on the outcome variables. Consequently, participants were excluded who did not fulfill the age criterion (N = 87,493), worked less than 12 h per week (N = 18,113), had missing data on baseline MetS components (N = 660), dropped out before the second comprehensive assessment (N = 9,150), or had missing data on follow-up MetS components (N = 2,439). Lastly, participants in the major occupational category “armed forces” were excluded (N = 39) as the skill level of this category varies and there are no tasks specified for this International Standard Classification of Occupations (ISCO) 08 (International Labour Office, 2012) category (Mihaylov & Tijdens, 2019). Therefore, it is difficult to categorize this group for the current analysis. The final study population consisted of N = 34,834 participants.

To analyze the incidence of MetS and its components, participants who classify for baseline MetS or the respective baseline MetS component were excluded. This resulted in the following sample sizes: overall MetS (N = 28,266), abdominal obesity (N = 21,861), raised triglycerides (N = 27,115), reduced HDL-cholesterol (N = 29,067), elevated blood pressure (N = 17,929), and elevated fasting glucose (N = 29,548).

### 2.2 Measures

#### 2.2.1 Metabolic syndrome (MetS)

Participants were classified as having MetS based on the joint interim criteria (Alberti et al., 2009). For a MetS diagnosis, three or more of the following five components need to be present: abdominal obesity (waist circumference ≥ 102 cm in males, waist circumference ≥ 88 cm in females), elevated triglycerides (≥1.7 mmol/L) or medical treatment for this lipid abnormality (Anatomical Therapeutic Chemical (ATC) code C10A or C10B) (WHO, 2019), reduced HDL-Cholesterol (<1.0 mmol/L in males, < 1.3 mmol/L in females) or medical treatment for this lipid abnormality (ATC code C10A or C10B) (WHO, 2019), elevated blood pressure (systolic ≥ 130 and/or diastolic ≥ 85 mm Hg) or hypertension treatment (ATC code C02, C03, C07, C08, or C09) (WHO, 2019), elevated fasting glucose (≥5.6 mmol/L) or medical treatment for type two diabetes mellitus (T2DM) (ATC codes A10A or A10B) (WHO, 2019). MetS components were measured by trained research staff using calibrated measuring equipment and standardized protocols (Scholtens et al., 2015). Waist circumference was assessed in an upright position and in the middle between the front end of the lower ribs and the iliac crest. Triglycerides, HDL-cholesterol, and fasting glucose were based on fasting blood samples. Systolic and diastolic blood pressure were measured by an automatic blood pressure monitor. ATC codes were recorded only at baseline.

#### 2.2.2 Occupational group membership

Occupational group membership was self-reported at baseline and automatically coded in line with the ISCO-08 (International Labour Office, 2012) by Statistics Netherlands (van Zon et al., 2020). The first nine of the ten major groups were included in the current study, i.e. (1) managers, (2) professionals, (3) technicians and associate professionals, (4) clerical support workers, (5) services and sales workers, (6) skilled agricultural forestry and fishery workers, (7) craft and related trades workers, (8) plant and machine operators and assemblers, and (9) elementary occupations. The nine groups were categorized into four major occupational categories: high skilled white-collar (groups 1–3), low skilled white-collar (groups 4–5), high skilled blue-collar (groups 6–7), and low skilled blue-collar (groups 8–9) (Brønnum-Hansen et al., 2020).

#### 2.2.3 Health behaviors

Health behaviors were self-reported at baseline and consisted of smoking status, leisure-time physical activity (LTPA), diet quality, and alcohol consumption. *Smoking status* was categorized into current-, ex-, or never smokers (van Zon et al., 2020). *LTPA* was assessed by the Short QUestionnaire to ASsess Health enhancing physical activity (SQUASH) which measures light, moderate, and vigorous active minutes per week in different domains (Wendel-Vos, Schuit, Saris, & Kromhout, 2003). According to the revised SQUASH data processing protocol, only moderate to vigorous active minutes per week during leisure time were analyzed instead of the total amount of physical activity as this is the recommended measure of habitual physical activity (Byambasukh and Corpeleijn, 2020, Byambasukh et al., 2020). LTPA was then coded into no (0 min per week), and low (1–120 min per week), medium (121–300 min per week), and high (301 or more minutes per week) tertiles (Byambasukh et al., 2020). *Diet Quality* concerned energy and macronutrient intake over the previous month, which was assessed by a food frequency questionnaire and then coded into the Lifelines Diet Score (LLDS) (Vinke et al., 2018). The LLDS was developed in line with the 2015 Dutch Dietary Guidelines (Kromhout, Spaaij, de Goede, & Weggemans, 2016) and differentiates diet quality between participants using quintiles ranging from low (1) to high (5). Participants in quintile 5 consume the highest rate of positive food groups (e.g. vegetables, fruits, or whole grain products) and the lowest rate of negative food groups (e.g. sugar-sweetened beverages or red and processed meats). More details on the LLDS can be found elsewhere (Vinke et al., 2018). *Weekly alcohol consumption* was self-reported and categorized into drinking 0 days, 0–1 days, >1–3 days, and >3 days (van Zon et al., 2020).

To analyze *combinations of unhealthy behaviors*, smoking status, LTPA, diet quality, and alcohol consumption were binary coded. Ex- or never-smokers were coded as “no smoking” and current smokers as “smoking”. Medium or high LTPA was categorized as “high LTPA” and low or no LTPA as “low LTPA”. Diet quintiles 3–5 were coded as “healthy diet” and diet quintiles 1–2 as “unhealthy diet”. Drinking alcohol on less than four days per week was coded as “low alcohol consumption” and drinking alcohol on four days or more per week was coded as “high alcohol consumption” (Ots et al., 2020).

#### 2.2.4 Sociodemographic factors

Age, gender, ethnicity, marital status, educational level, and weekly working hours were self-reported at baseline. *Age and gender* were included as covariates due to the age-associated increase and different effects among males and females regarding MetS prevalence and incidence (Li et al., 2018; van Zon et al., 2020). *Ethnicity* was categorized into White: East and West European, White: Mediterranean/Arabic, Black, Asian, or Other. *Marital status* was categorized into married, partner with cohabitation, partner without cohabitation, no partner, or other. Marital status was included as a covariate because of a possible association with MetS (Erem et al., 2008). *Educational level* was categorized into low (no education; primary education; lower or preparatory secondary vocational education; junior general secondary education), medium (secondary vocational education or work-based learning; senior general secondary education, pre-university secondary education), high (higher vocational education; university education), or other (Ots et al., 2020). *Weekly working hours* were categorized into working 12–19 hours, 20–31 hours, 32–40 hours, or more than 40 hours, and included as a covariate to adjust for the potential effect of work environment on MetS incidence (Watanabe et al., 2018). *Follow-up time* in months between baseline assessment and the second comprehensive assessment was included as a covariate to account for varying follow-up times of participants.

#### 2.2.5 Multiple imputation

Multiple imputation was performed to deal with missing data on the key study variables (White, Royston, & Wood, 2011). Participants who dropped out before the second comprehensive assessment (N = 9,150), and with missing values on outcome variables, i.e. baseline MetS components (N = 660) and follow-up MetS components (N = 2,439), were excluded before multiple imputation. Consequently, data were missing for 18.7% of participants (N = 6,516) and it was imputed on the total baseline study population (N = 34,834) (Table 1). Specifically, missing values were imputed on marital status (N = 12), occupational group (N = 945), smoking status (N = 198), LTPA (N = 2,423), diet quality (N = 3,734), and alcohol consumption (N = 221). The imputations were predicted by follow-up time, age, sex, marital status, occupational group, weekly working hours, smoking status, LTPA, diet quality, follow-up MetS, and follow-up MetS components. Multiple imputation of missing data was performed 20 times following the recommendation to impute at least as often as the percentage of incomplete cases (White et al., 2011). Pooled results are displayed for the logistic regression analyses and descriptive statistics are taken from one of the multiple imputed datasets. IBM SPSS Statistics version 25 was used to conduct multiple imputation.

**Table 1:** Baseline Characteristics of the Study Population (N = 34,834).

	N	% or mean (SD)	Missing values (%)
Sociodemographic factors			
Age, years	34,834	51.2 (5.0)	0
Sex			0
Female	18,488	53.1	
Male	16,346	46.9	
Ethnicity			7.2
White: East and West European	31,884	91.5	
White: Mediterranean/Arabic	63	0.2	
Black	35	0.1	
Asian	139	0.4	
Other	216	0.6	
Marital status			0
Married	26,959	77.4	
Partner, cohabitation	3,299	9.5	
Partner, no cohabitation	1,058	3.0	
No partner	2,007	5.8	
Other	1,499	4.3	
Educational level			0.1
High	10,587	30.4	
Medium	13,131	37.7	
Low	10,501	30.1	
Other	589	1.7	
Occupational group			2.7
High skilled white-collar	17,022	48.9	
Low skilled white-collar	10,483	30.0	
High skilled blue-collar	3,559	10.2	
Low skilled blue-collar	2,870	8.2	
Weekly working hours			0
>40 hours	5,543	15.9	
32–40 hours	15,401	44.2	
20–31 hours	9,743	28.0	
12–19 hours	4,147	11.9	
**Health Behaviors**			
Smoking status			0.6
Never smoker	13,953	40.1	
Current smoker	5,975	17.2	
Ex-smoker	14,708	42.2	
LTPA			7.0
High	7,748	22.2	
Medium	9,205	26.4	
Low	9,557	27.4	
None	5,901	16.9	
Diet quintile			10.7
5 (healthiest)	7,274	20.9	
4	7,580	21.8	
3	6,127	17.6	
2	6,200	17.8	
1	3,919	11.3	
Weekly alcohol consumption			0.6
0 days	5,749	16.5	
0–1 days	9,933	28.5	
>1–3 days	9,013	25.9	
>3 days	9,918	28.5	
**Health**			
MetS	6,568	18.9	0
MetS components			0
Abdominal obesity	12,973	37.2	
Raised triglycerides	7,719	22.2	
Reduced HDL cholesterol	5,767	16.6	
Elevated blood pressure	16,905	48.5	
Elevated fasting glucose	5,286	15.2	

*Note*: SD, standard deviation; LTPA, leisure-time physical activity; MetS, metabolic syndrome; HDL, high-density lipoprotein.

#### 2.2.6 Statistical analyses

First, the distribution of baseline characteristics and the distribution of health behaviors by occupational group membership were assessed using descriptive statistics for the baseline study population. Second, incidence rates of MetS and its individual components were examined by sociodemographic factors and health behaviors. Third, logistic regression analysis was performed to examine the association between occupational group membership and MetS incidence. This analysis was adjusted for age, gender, marital status, weekly working hours, and follow-up time (model 1). Educational level was not included in the regression analyses due to the assumed conceptual overlap with occupational group membership (van Zon et al., 2020). Fourth, smoking, LTPA, diet quality, and alcohol consumption were added to model 1 to examine to what extent the differences in MetS incidence can be explained by health behaviors (model 2). Odds ratio's (ORs) and 95 % confidence intervals (CIs) were computed. These analyses were repeated for the individual MetS components. Fifth, the prevalence of combinations of unhealthy behaviors was examined across occupational groups using descriptive statistics. Finally, the association between combinations of unhealthy behaviors and MetS incidence was examined using logistic regression analysis. IBM SPSS Statistics version 25 was used to perform the data analyses.

## 3 Results

### 3.1 Sample characteristics

The study sample consisted of N = 34,834 participants with a mean age of 51.2 years (SD: 5.0) (Table 1). Most of the participants were high skilled white-collar workers (48.9 %) and least were low skilled blue-collar workers (8.2 %). The majority of participants were ex-smokers (42.2 %) or never-smokers (40.1 %). Most participants indicated low LTPA (27.4 %) or medium LTPA (26.4 %) and were grouped either in the healthiest diet quintile 5 (20.9 %) or quintile 4 (21.8 %). Most participants indicated to drink alcohol on >3 days per week (28.5 %) or 0–1 days per week (28.5 %). Except for high alcohol consumption, unhealthier behaviors were more prevalent in blue-collar workers than white-collar workers (Supplemental Table 1). High-skilled white-collar workers indicated higher weekly alcohol consumption than the other occupational groups. Lastly, the most prevalent MetS component at baseline was elevated blood pressure (48.5 %) (Table 1). Compared to the study sample, participants who dropped out before the second comprehensive assessment were more often blue-collar workers, had a slightly higher prevalence of unhealthy behaviors and were overall somewhat unhealthier (Supplemental Table 2). The mean follow-up time was 3.85 years (SD: 1.10).

### 3.2 MetS incidence by occupational group membership and health behaviors

In total, N = 2,419 (8.6 %) participants developed MetS. MetS incidence was lowest among high skilled white-collar workers (7.9 %) and increased with every consecutive lower occupational group up to 10.7 % among low skilled blue-collar workers (Table 2). Except for alcohol consumption, MetS incidence was lowest among the groups with the healthiest behaviors, i.e. never smokers (7.5 %), high LTPA (6.8 %), or diet quintile 5 (6.7 %) and highest in the unhealthiest groups, i.e. current smokers (11.2 %), no LTPA (11.8 %), and diet quintile 1 (10.9 %). MetS incidence was highest in participants who indicated no alcohol consumption (9.9 %) and lowest among participants who indicated to drink >3 days per week (7.9 %). Low skilled white-collar (adjusted OR: 1.24; 95 % CI: 1.12, 1.37), and low skilled blue-collar workers (adjusted OR: 1.37; 95 % CI: 1.18, 1.59) had a significantly higher MetS incidence rate compared to high skilled white-collar workers (model 1). After adding health behaviors (model 2), the association between occupational group membership and MetS incidence was attenuated but remained statistically significant among low skilled white-collar workers (adjusted OR: 1.13; 95 % CI: 1.02, 1.25) (Table 2). Among low skilled blue-collar workers, the association was attenuated and no longer significant (model 2).

**Table 2:** Baseline Risk Factors and MetS Incidence – Results of a Logistic Regression Analysis^a^ (N = 28,266).

	N MetS/N Total	%	Model 1	Model 2
			OR (95 % CI)	OR (95 % CI)
**Sociodemographic Factors**				
Follow-up time, months			1.00 (1.00, 1.01)	1.00 (1.00, 1.00)
Age, years			1.01 (1.00, 1.02)	**1.01 (1.00, 1.02)**
Sex				
Female	1,116/15,780	7.1	Ref.	
Male	1,303/12,486	10.4	**1.53 (1.37, 1.72)**	**1.58 (1.40, 1.78)**
Marital status				
Married	1,866/21,812	8.6	Ref.	
Partner, cohabitation	213/2761	7.7	0.92 (0.80, 1.07)	0.91 (0.79, 1.06)
Partner, no cohabitation	75/884	8.5	1.04 (0.82, 1.33)	1.02 (0.80, 1.30)
No partner	143/1612	8.9	1.09 (0.91, 1.31)	1.06 (0.89, 1.27)
Other			**1.31 (1.09, 1.57)**	**1.23 (1.01, 1.50)**
Occupational group				
High skilled white-collar	1,146/14,480	7.9	Ref.	
Low skilled white-collar	762/8706	8.8	**1.24 (1.12, 1.37)**	**1.13 (1.02, 1.25)**
High skilled blue-collar	271/2846	9.5	1.03 (0.89, 1.20)	0.92 (0.79, 1.07)
Low skilled blue-collar	240/2234	10.7	**1.37 (1.18, 1.59)**	1.16 (1.00, 1.36)
Weekly working hours				
>40 hours	441/4373	10.1	1.04 (0.93, 1.17)	1.03 (0.92, 1.16)
32–40 hours	1,133/12,243	9.3	Ref.	
20–31 hours	573/8242	7.0	0.91 (0.80, 1.02)	0.92 (0.81, 1.04)
12–19 hours	272/3408	8.0	1.01 (0.86, 1.19)	1.02 (0.87, 1.20)
**Health Behaviors**				
Smoking Status				
Never smoker	893/11,879	7.5		Ref.
Current smoker	524/4682	11.2		**1.48 (1.31, 1.66)**
Ex-smoker	1,002/11,705	8.6		**1.23 (1.11, 1.35)**
LTPA				
High	511/7489	6.8		Ref.
Medium	651/8339	7.8		**1.18 (1.04, 1.34)**
Low	743/8073	9.2		**1.35 (1.19, 1.52)**
None	514/4365	11.8		**1.65 (1.44, 1.88)**
Diet quintile				
5 (healthiest)	451/6753	6.7		Ref.
4	549/7022	7.8		1.09 (0.95, 1.26)
3	495/5554	8.9		**1.21 (1.05, 1.39)**
2	554/5543	10.0		**1.27 (1.10, 1.47)**
1	370/3394	10.9		**1.36 (1.16, 1.60)**
Weekly alcohol consumption				
0 days	446/4522	9.9		Ref.
0–1 days	728/8107	9.0		**0.85 (0.75, 0.96)**
>1–3 days	596/7436	8.0		**0.71 (0.62, 0.81)**
>3 days	649/8201	7.9		**0.66 (0.58, 0.76)**

*Note*: MetS, metabolic syndrome; OR, odds ratio; CI, confidence interval; Ref., reference group; LTPA, leisure-time physical activity.

a OR's written in bold are significant (P < .05).

**Fig. 1:**
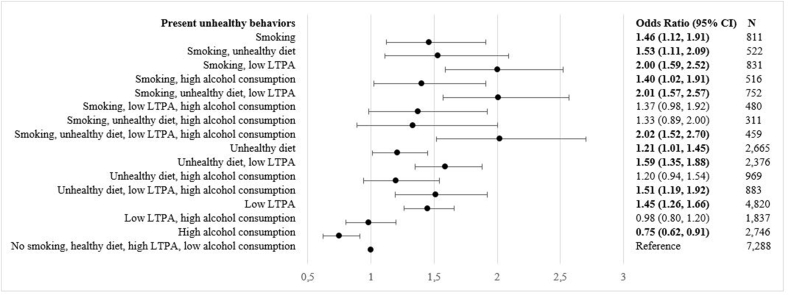
Health Behavior Combinations and MetS Incidence – Results of a Logistic Regression Analysis^a^ (N = 28,266) *Note*: MetS, metabolic syndrome; CI, confidence interval; LTPA, leisure-time physical activity. OR's written in bold are significant (P < .05). ^a^Adjusted for age, sex, follow-up time, marital status, and working hours.

As unhealthy behaviors often cluster, the prevalence of combinations of unhealthy behaviors was examined by occupational group (Table 3). Overall, blue-collar workers had a higher rate of engaging in combinations of smoking, unhealthy diet, and low LTPA than white-collar workers. For instance, 12.0 % of low skilled blue-collar workers and 14.0 % of high skilled blue-collar workers engaged in a combination of unhealthy diet and low LTPA, compared to 6.4 % of high skilled white-collar workers. High alcohol consumption alone was most prevalent among high skilled white-collar workers (12.6 %). Further, high skilled white-collar workers had the highest prevalence of healthy behaviors (i.e. no smoking, healthy diet, high LTPA, low alcohol consumption) (27.3 %) whereas high skilled blue-collar workers had the lowest prevalence (18.8 %). Compared to participants without any unhealthy behaviors, MetS incidence was highest among participants who engaged all four unhealthy behaviors (adjusted OR: 2.02; 95 % CI: 1.52, 2.70) (Fig. 1).

**Table 3:** Health Behavior Combinations by Occupational Group (N = 28,266).

				Occupational Group				
				High skilled white-collar	Low skilled white-collar	High skilled blue-collar	Low skilled blue-collar	N
				%	%	%	%	
**Health Behavior Combinations**								
**Smoking**	**Unhealthy Diet**	**Low LTPA**	**High Alcohol**					
No	No	No	No	27.3	26.5	18.8	22.3	7,288
Yes	No	No	No	2.7	3.4	2.0	3.4	811
No	Yes	No	No	8.3	9.2	14.4	11.1	2,665
No	No	Yes	No	16.5	18.8	15.2	16.2	4,820
No	No	No	Yes	12.6	7.5	5.7	4.7	2,746
Yes	Yes	No	No	1.3	2.2	2.5	3.1	522
No	Yes	Yes	No	6.4	8.9	14.0	12.0	2,376
No	No	Yes	Yes	8.0	5.5	4.5	3.4	1,837
Yes	No	Yes	No	2.3	3.5	2.7	5.0	831
Yes	No	No	Yes	2.1	1.7	0.8	1.5	516
No	Yes	No	Yes	3.7	2.9	3.9	2.9	969
Yes	Yes	Yes	No	1.6	3.3	3.9	5.6	752
Yes	No	Yes	Yes	1.8	1.8	1.5	1.2	480
Yes	Yes	No	Yes	1.0	0.9	1.8	1.5	311
No	Yes	Yes	Yes	2.9	2.5	5.4	3.7	883
Yes	Yes	Yes	Yes	1.4	1.4	2.8	2.4	459
Total				100	100	100	100	28,266

*Note*: LTPA, leisure-time physical activity.

### 3.3 Incidence of MetS components by occupational group membership

Generally, the analyses on the MetS component level showed similar patterns as the analyses on MetS itself, i.e. the incidence risk of any component is highest among low skilled blue-collar workers (Supplemental Table 3). However, the incidence rates of the components differ, i.e. the highest incidence rate is observed for raised blood pressure (N = 5,032, 28.1 %), followed by abdominal obesity (N = 3,140, 14.4 %), raised triglycerides (N = 2,591, 9.6 %), raised blood glucose (N = 2,746, 9.3 %), and lastly reduced HDL-cholesterol (N = 1,470, 5.1 %). The overall highest incidence risk was observed among low skilled blue-collar workers for abdominal obesity (adjusted OR: 1.70; 95 % CI: 1.47, 1.96).

After adding health behaviors to model 1, the effect of occupational groups on incident low HDL-cholesterol was attenuated and no longer significant (Supplemental Table 3). Smoking, unhealthier diet, and lower LTPA were significantly related to all MetS components, with the highest ORs observed among the unhealthiest groups. Higher alcohol consumption was associated with a significantly lower incidence risk of all MetS components.

## 4 Discussion

In this 3.85-year follow-up study among N = 34,834 older workers from the Netherlands, occupational group membership was associated with MetS incidence. Low skilled workers, particularly in blue-collar occupations, had the highest MetS incidence rate (10.7 %). Health behaviors explained a substantial part of the MetS incidence differences among occupational groups. Results were similar for the incidence of the individual MetS components. Especially low skilled blue-collar workers were at increased risk for incident abdominal obesity, the major driving MetS component (Eckel et al., 2005).

Substantial occupational differences in MetS incidence, and its components, were found among older workers. This is the first study to investigate the incidence of MetS specifically among the aging workforce and thereby adds important information on MetS incidence among older workers in different occupational groups. Previous longitudinal studies about MetS among workers of all ages also showed a higher MetS incidence risk in blue-collar occupations like food preparation assistants and plant and machine operators (van Zon et al., 2020), and a lower MetS incidence risk among individuals with a higher socioeconomic position (Hoveling, Liefbroer, Bültmann, & Smidt, 2021).

Smoking, unhealthy diet, and low LTPA were associated with a higher MetS and MetS components incidence risk and clustered specifically among blue-collar workers. In contrast, higher alcohol consumption was associated with a lower MetS and MetS components incidence risk and was more common among high skilled white-collar workers. The finding that unhealthy behaviors generally cluster among older workers in blue-collar occupations aligns with recent studies among the general working population (Thebault et al., 2018; Väisänen et al., 2020).

The result that higher alcohol consumption was associated with a lower risk of MetS and MetS components incidence might be explained by two reasons. First, previous literature shows that a moderate consumption of beer and wine can have a protective effect against MetS whereas the opposite is seen for liquor or spirit consumption and binge drinking (Pérez-Martínez et al., 2017). The positive effect of higher alcohol consumption on MetS in this study might be related to the unmeasured amount of alcohol per drinking session and type of alcohol consumed. Second, the reference group “drinking 0 days per week”, with the highest MetS incidence rate, might include unhealthier participants than in the other drinking groups who cannot drink alcohol because of medication use or pre-existing health conditions. Future research would benefit from using a more precise measure of alcohol consumption and taking underlying health conditions into account.

After adjusting for baseline health behaviors, still around half of the occupational differences in MetS incidence remained unexplained. There are several explanations for this finding. First, there might be other factors at play than the health behaviors included in this study. For instance, occupational physical activity, which is more common among blue-collar workers than white-collar workers, might mask the positive health effect of LTPA (Holtermann, Krause, van der Beek, & Straker, 2018). In contrast to LTPA, occupational physical activity may have a detrimental health effect which has been described as the physical activity paradox (Holtermann et al., 2018). Second, not the occupation per se but an adverse psychosocial work environment and related consequences might be important for MetS incidence. For example, psychosocial stress at work has a detrimental influence on MetS (Watanabe et al., 2018) and persistent work stress has been shown to accumulate over the life-course which can lead to chronic negative health outcomes later in life (Igic et al., 2017). Third, health behaviors earlier in life might influence the MetS incidence later in the working life. Unhealthy behaviors tend to accumulate over the working life course especially in blue-collar workers (Lynch et al., 1997). It is possible that the detrimental health effect of unhealthy behaviors earlier in life might have already partly played their effect out on the MetS incidence of older workers and that baseline health behaviors do not fully capture these health careers. This aligns with the fact that many older workers (N = 6,568, 18.9 %) already had MetS at baseline assessment.

The study has several strengths. The risk of information bias is limited due to the methodological quality of the data. MetS diagnosis was based on a combination of physical examinations, blood analyses and medication use, obtained by trained research staff (van Zon et al., 2020). LTPA and diet quality were measured by extensive validated questionnaires (Vinke et al., 2018; Wendel-Vos et al., 2003). Occupational group membership was coded by Statistics Netherlands, and underwent rigorous quality control (van Zon et al., 2020). Moreover, the use of longitudinal data reduces the risk of reverse causation. Finally, Lifelines is representative for the Northern part of the Netherlands, which facilitates the generalizability of results (Klijs et al., 2015).

There are also limitations to be taken into account. First, no life-course information about the effect of earlier health behaviors, occupational careers, and psychosocial work environment factors were included. Second, N = 9,150 (19.7 %) of participants dropped out before the second comprehensive assessment which might have resulted in some selection bias. Descriptive statistics showed that the excluded workers were more often blue-collar workers and slightly unhealthier than the included workers, but differences were rather small. Third, the change in odds ratios attributed to adding health behaviors to the logistic regression model is not an absolute measure but an approximation of the effect of health behaviors on occupational MetS incidence differences. Different odds ratios from the same study cannot be compared in absolute terms when different explanatory variables are used in the models that are compared to each other (Norton, Dowd, & Maciejewski, 2018).

The results of this study may have some implications for clinical practice and policy makers. First, individual motivation to change towards a healthier lifestyle – though difficult – has been described as a major factor in MetS reduction which can be enhanced by personal contact with health care workers and the use of technology (Bassi et al., 2014). Health behaviors explained a substantial part of MetS incidence among this sample of older workers, but a promotion of a healthy lifestyle might be needed already earlier in the working life to prevent the incidence of MetS and its components later on. Second, workers and employers might not be aware of MetS incidence among older workers. Therefore, contact with the (occupational) health care system might be beneficial for MetS prevention (Bassi et al., 2014). Regular health check-ups by occupational physicians at the workplace could raise awareness of MetS and underline the need of MetS prevention. A holistic approach is needed to promote a healthy lifestyle at work among all occupational groups, and especially among blue-collar occupations, which pays attention to barriers at the workplace and to the broader context of blue-collar workers (Lingard & Turner, 2015).

The results of this study also have some implications for future research. First, life-course studies are needed to determine the possible cumulative influence of early life health behaviors and work history on later life health (dis-)advantages (Amick, McLeod, & Bültmann, 2016). Further, future research should include longer and repeated follow-up data from several assessment waves and take the influence of changes in health behaviors over time into account. Additionally, other potential risk factors like the psychosocial work environment (Watanabe et al., 2018) and occupational physical activity (Brighenti-Zogg et al., 2016) should be investigated over the working life-course and among older workers. MetS has been related to early death after retirement (Sakurai et al., 2020) and possibly lower quality of life in retirement due to MetS related consequences like CVD and T2DM (Laiteerapong et al., 2011; Schofield et al., 2012), thus it is important to examine the effect of the retirement transition on MetS and its components. It is currently unclear how the retirement transition influences objective health outcomes like MetS (Xue, Head, & McMunn, 2020). Finally, studies in various ethnic groups are needed as risks for poor cardio metabolic health outcomes differ between ethnic groups (Wells, 2012).

In conclusion, occupational group membership matters for MetS incidence, and its components, among the aging workforce. Especially low skilled workers seem to be at risk for MetS. Smoking, low LTPA, an unhealthier diet, and higher alcohol consumption explained more of the overall MetS risk among low skilled blue-collar workers than in the other occupational groups. Consequently, older low skilled blue-collar workers might especially profit from targeted health promotion interventions and organizational changes that facilitate a healthier lifestyle.

## CRediT authorship contribution statement

**Katharina Runge:** Conceptualization, Data curation, Formal analysis, Methodology, Project administration, Validation, Visualization, Writing – original draft. **Sander K.R. van Zon:** Conceptualization, Methodology, Supervision, Validation, Writing – review & editing. **Ute Bültmann:** Conceptualization, Methodology, Supervision, Validation, Writing – review & editing. **Kène Henkens:** Conceptualization, Methodology, Supervision, Validation, Writing – review & editing.

## Declaration of competing interest

None.
