# Supplementary material for: Metabolic syndrome incidence in an aging workforce: Occupational differences and the role of health behaviors
Source: SSM Popul Health. 2021 Jul 28;15:100881. doi: 10.1016/j.ssmph.2021.100881 (PMC8350497; doi:10.1016/j.ssmph.2021.100881)
Supplement: Multimedia component 1 [file mmc1.docx]

**Supplemental Table 1: Health Behavior Percentages by Occupational Group (N=28,266)**

|  | **Occupational group** | | | | | | | |
| --- | --- | --- | --- | --- | --- | --- | --- | --- |
|  | High skilled white-collar | | Low skilled white-collar | | High skilled blue-collar | | Low skilled blue-collar | |
|  | N | % | N | % | N | % | N | % |
| **Smoking Status** |  |  |  |  |  |  |  |  |
| Never smoker | 6,504 | 44.9 | 3,294 | 37.8 | 1,296 | 45.5 | 785 | 35.1 |
| Current smoker | 2,057 | 14.2 | 1,586 | 18.2 | 514 | 18.1 | 525 | 23.5 |
| Ex-smoker | 5,919 | 40.9 | 3,826 | 43.9 | 1,036 | 36.4 | 924 | 41.4 |
| **LTPA** |  |  |  |  |  |  |  |  |
| High | 3,993 | 27.6 | 2,208 | 25.4 | 718 | 25.2 | 570 | 25.5 |
| Medium | 4.552 | 31.4 | 2,527 | 29.0 | 703 | 24.7 | 557 | 24.9 |
| Low | 4,102 | 28.3 | 2,555 | 29.3 | 804 | 28.3 | 612 | 27.4 |
| None | 1,833 | 12.7 | 1,416 | 16.3 | 621 | 21.8 | 495 | 22.2 |
| **Diet Quintile** |  |  |  |  |  |  |  |  |
| 5 (healthiest) | 4,068 | 28.1 | 2,001 | 23.0 | 327 | 11.5 | 357 | 16.0 |
| 4 | 3,798 | 26.2 | 2,208 | 25.4 | 556 | 19.5 | 460 | 20.6 |
| 3 | 2,734 | 18.9 | 1,772 | 20.4 | 575 | 20.2 | 473 | 21.2 |
| 2 | 2,488 | 17.2 | 1,726 | 19.8 | 780 | 27.4 | 549 | 24.6 |
| 1 | 1,392 | 9.6 | 999 | 11.5 | 608 | 21.4 | 395 | 17.7 |
| **Weekly alcohol consumption** |  |  |  |  |  |  |  |  |
| 0 days | 1,899 | 13,1 | 1,817 | 20.9 | 314 | 11.0 | 492 | 22.0 |
| 0-1 days | 3,840 | 26.5 | 2,719 | 31.2 | 870 | 30.6 | 678 | 30.3 |
| >1-3 days | 3,880 | 26.8 | 2,057 | 23.6 | 911 | 32.0 | 588 | 26.3 |
| >3 days | 4,861 | 33.6 | 2,113 | 24.3 | 751 | 26.4 | 476 | 21.3 |

*Note*: LTPA, leisure-time physical activity.

**Supplemental Table 2: Baseline Characteristics of the Study Population and Drop-Out Sample**

|  | **Study population** | | **Drop-out sample** | |
| --- | --- | --- | --- | --- |
|  | **N** | **% or mean (SD)** | **N** | **% or mean (SD)** |
| **Sociodemographic factors** |  |  |  |  |
| Age, years | 34,834 | 51.2 (5.0) | 9,150 | 50.1 (4.6) |
| Sex |  |  |  |  |
| Female | 18,488 | 53.1 | 4,711 | 51.5 |
| Male | 16,346 | 46.9 | 4,439 | 48.5 |
| Ethnicity |  |  |  |  |
| White: East and West  European | 31,884 | 91.5 | 5,873 | 97.4 |
| White: Mediterranean /  Arabic | 63 | 0.2 | 30 | 0.5 |
| Black | 35 | 0.1 | 15 | 0.2 |
| Asian | 139 | 0.4 | 39 | 0.6 |
| Other | 216 | 0.6 | 72 | 1.2 |
| Marital status |  |  |  |  |
| Married | 26,959 | 77.4 | 6,831 | 74.7 |
| Partner, cohabitation | 3,299 | 9.5 | 1,014 | 11.1 |
| Partner, no cohabitation | 1,058 | 3.0 | 307 | 3.4 |
| No partner | 2,007 | 5.8 | 550 | 6.0 |
| Other | 1,499 | 4.3 | 445 | 4.9 |
| Educational level |  |  |  |  |
| High | 10,587 | 30.4 | 2,198 | 24.0 |
| Medium | 13,131 | 37.7 | 3,442 | 37.7 |
| Low | 10,501 | 30.1 | 3,333 | 36.5 |
| Other | 589 | 1.7 | 168 | 1.8 |
| Occupational group |  |  |  |  |
| High skilled white-collar | 17,022 | 48.9 | 3,843 | 43.5 |
| Low skilled white-collar | 10,483 | 30.0 | 2,890 | 32.7 |
| High skilled blue-collar | 3,559 | 10.2 | 1,083 | 12.3 |
| Low skilled blue-collar | 2,870 | 8.2 | 1,016 | 11.5 |
| Weekly working hours |  |  |  |  |
| >40 hours | 5,543 | 15.9 | 1,653 | 18.1 |
| 32-40 hours | 15,401 | 44.2 | 4,091 | 44.7 |
| 20-31 hours | 9,743 | 28.0 | 2,372 | 25.9 |
| 12-19 hours | 4,147 | 11.9 | 1,034 | 11.3 |
| **Health Behaviors** |  |  |  |  |
| Smoking status |  |  |  |  |
| Never smoker | 13,953 | 40.1 | 3,294 | 37.8 |
| Current smoker | 5,975 | 17.2 | 2,182 | 25.1 |
| Ex-smoker | 14,708 | 42.2 | 3,229 | 37.1 |
| LTPA |  |  |  |  |
| High | 7,748 | 22.2 | 1,717 | 21.6 |
| Medium | 9,205 | 26.4 | 1,921 | 24.1 |
| Low | 9,557 | 27.4 | 2,307 | 29.0 |
| None | 5,901 | 16.9 | 2,015 | 25.3 |
| Diet quintile |  |  |  |  |
| 5 (healthiest) | 7,274 | 20.9 | 1,490 | 18.9 |
| 4 | 7,580 | 21.8 | 1,660 | 21.0 |
| 3 | 6,127 | 17.6 | 1,533 | 19.4 |
| 2 | 6,200 | 17.8 | 1,818 | 23.0 |
| 1 | 3,919 | 11.3 | 1,397 | 17.7 |
| Weekly alcohol consumption |  |  |  |  |
| 0 days | 5,749 | 16.5 | 1,619 | 18.7 |
| 0-1 days | 9,933 | 28.5 | 2,558 | 29.6 |
| >1-3 days | 9,013 | 25.9 | 2,138 | 24.8 |
| >3 days | 9,918 | 28.5 | 2,320 | 26.9 |
| **Health** |  |  |  |  |
| MetS | 6,568 | 18.9 | 2,115 | 23.1 |
| MetS components |  |  |  |  |
| Abdominal obesity | 12,973 | 37.2 | 3,965 | 43.3 |
| Raised triglycerides | 7,719 | 22.2 | 2,302 | 25.2 |
| Reduced HDL-cholesterol | 5,767 | 16.6 | 1,763 | 19.3 |
| Elevated blood pressure | 16,905 | 48.5 | 4,817 | 52.6 |
| Elevated fasting glucose | 5,286 | 15.2 | 1,636 | 17.9 |

*Note*: SD, standard deviation; LTPA, leisure-time physical activity; MetS, metabolic syndrome; HDL, high-density lipoprotein.

**Supplemental Table 3: Baseline Risk Factors and Incidence of MetS Components – Results of Logistic Regression Analyses**^a^

|  | **Abdominal obesity (N=21,861)** | | **Raised triglycerides (N=27,115)** | | **Red. HDL-cholesterol (N=29,067)** | | **Raised blood pressure (N=17,929)** | | **Raised fasting glucose (N=29,548)** | |
| --- | --- | --- | --- | --- | --- | --- | --- | --- | --- | --- |
|  | **Model 1** | **Model 2** | **Model 1** | **Model 2** | **Model 1** | **Model 2** | **Model 1** | **Model 2** | **Model 1** | **Model 2** |
|  | OR (95% CI) | OR (95% CI) | OR (95% CI) | OR (95% CI) | OR (95% CI) | OR (95% CI) | OR (95% CI) | OR (95% CI) | OR (95% CI) | OR (95% CI) |
| **Sociodemographic Factors** |  |  |  |  |  |  |  |  |  |  |
| Follow-up time, months | **1.01 (1.00, 1.01)** | **1.01 (1.00, 1.01)** | 1.00 (1.00, 1.00) | 1.00 (1.00, 1.00) | **0.99 (0.99, 1.00)** | **0.99 (0.99, 1.00)** | **1.00 (1.00, 1.01)** | **1.00 (1.00, 1.01)** | **1.01 (1.01, 1.01)** | **1.01 (1.01, 1.01)** |
| Age, years | **1.02 (1.01, 1.02)** | **1.02 (1.01, 1.03)** | **0.99 (0.98, 1.00)** | 1.00 (0.99, 1.00) | **0.98 (0.97, 0.99)** | 0.99 (0.98, 1.00) | **1.05 (1.04, 1.05)** | **1.05 (1.04, 1.06)** | **1.02 (1.01, 1.03)** | **1.02 (1.01, 1.03)** |
| Sex |  |  |  |  |  |  |  |  |  |  |
| Female | Ref. |  | Ref. |  | Ref. |  | Ref. |  | Ref. |  |
| Male | **0.49 (0.44, 0.55)** | **0.49 (0.44, 0.54)** | **2.26 (2.02, 2.53)** | **2.27 (2.02, 2.55)** | 1.06 (0.92, 1.22) | 1.10 (0.95, 1.27) | **1.60 (1.46, 1.74)** | **1.60 (1.46, 1.75)** | **2.03 (1.82, 2.27)** | **1.99 (1.78, 2.22)** |
| Marital status |  |  |  |  |  |  |  |  |  |  |
| Married | Ref. |  | Ref. |  | Ref. |  | Ref. |  | Ref. |  |
| Partner, cohabitation | 0.91 (0.79, 1.04) | 0.91 (0.80, 1.04) | 1.01 (0.88, 1.17) | 0.99 (0.86, 1.14) | 1.15 (0.97, 1.37) | 1.15 (0.96, 1.36) | 1.02 (0.91, 1.14) | 1.02 (0.91, 1.14) | 1.00 (0.87, 1.14) | 0.98 (0.85, 1.13) |
| Partner, no cohabitation | 0.90 (0.72, 1.13) | 0.89 (0.71, 1.12) | 1.17 (0.93, 1.47) | 1.11 (0.89, 1.40) | 1.13 (0.85, 1.49) | 1.12 (0.83, 1.50) | 0.83 (0.69, 1.01) | 0.83 (0.68, 1.01) | 1.05 (0.83, 1.32) | 1.01 (0.80, 1.28) |
| No partner | 1.12 (0.95, 1.31) | 1.11 (0.95, 1.31) | 1.00 (0.83, 1.20) | 0.98 (0.81, 1.18) | 1.19 (0.96, 1.47) | 1.12 (0.90, 1.39) | 0.92 (0.79, 1.06) | 0.91 (0.78, 1.05) | **1.36 (1.16, 1.60)** | **1.35 (1.15, 1.59)** |
| Other | 0.96 (0.79, 1.16) | 0.92 (0.76, 1.12) | **1.45 (1.20, 1.74)** | **1.35 (1.12, 1.63)** | **1.30 (1.15, 1.47)** | 1.21 (0.95, 1.55) | 1.16 (0.99, 1.35) | 1.13 (0.97, 1.33) | **1.29 (1.07, 1.55)** | **1.23 (1.02, 1.48)** |
| Occupational group |  |  |  |  |  |  |  |  |  |  |
| High skilled WC | Ref. |  | Ref. |  | Ref. |  | Ref. |  | Ref. |  |
| Low skilled WC | **1.21 (1.11, 1.33)** | **1.11 (1.02, 1.22)** | **1.21 (1.09, 1.33)** | **1.11 (1.00, 1.23)** | **1.15 (1.02, 1.31)** | 1.04 (0.91, 1.18) | **1.10 (1.01, 1.19)** | 1.06 (0.98, 1.15) | **1.22 (1.11, 1.34)** | **1.15 (1.04, 1.27)** |
| High skilled BC | **1.23 (1.07, 1.42)** | 1.13 (0.98, 1.31) | 1.04 (0.91, 1.20) | 0.94 (0.82, 1.08) | 0.98 (0.81, 1.19) | 0.86 (0.71, 1.05) | 1.00 (0.88, 1.13) | 0.96 (0.84, 1.08) | 0.98 (0.86, 1.12) | 0.91 (0.80, 1.05) |
| Low skilled BC | **1.70 (1.47, 1.96)** | **1.49 (1.29, 1.73)** | **1.36 (1.18, 1.57)** | **1.17 (1.01, 1.36)** | 1.20 (0.99, 1.46) | 1.01 (0.83, 1.23) | **1.28 (1.13, 1.46)** | **1.21 (1.06, 1.37)** | **1.26 (1.09, 1.45)** | 1.13 (0.97, 1.31) |
| Weekly working hours |  |  |  |  |  |  |  |  |  |  |
| >40 hours | 0.97 (0.86, 1.10) | 0.98 (0.86, 1.10) | 0.89 (0.79, 1.00) | **0.88 (0.78, 0.99)** | 1.03 (0.89, 1.20) | 1.02 (0.88, 1.19) | 1.05 (0.95, 1.16) | 1.04 (0.94, 1.15) | 1.02 (0.92, 1.14) | 1.02 (0.91, 1.14) |
| 32-40 hours | Ref. |  | Ref. |  | Ref. |  | Ref. |  | Ref. |  |
| 20-31 hours | 1.00 (0.90, 1.11) | 1.01 (0.91, 1.13) | 1.09 (0.96, 1.23) | 1.10 (0.98, 1.24) | 0.96 (0.82, 1.11) | 0.97 (0.83, 1.13) | 0.98 (0.90, 1.08) | 0.99 (0.90, 1.08) | 0.94 (0.83, 1.05) | 0.94 (0.84, 1.06) |
| 12-19 hours | 0.91 (0.79, 1.04) | 0.92 (0.80, 1.06) | 1.06 (0.90, 1.24) | 1.07 (0.91, 1.26) | 0.99 (0.81, 1.20) | 0.97 (0.80, 1.19) | 1.04 (0.92, 1.18) | 1.04 (0.93, 1.18) | 0.88 (0.75, 1.03) | 0.88 (0.75, 1.04) |
| **Health Behaviors** |  |  |  |  |  |  |  |  |  |  |
| Smoking Status |  |  |  |  |  |  |  |  |  |  |
| Never smoker |  | Ref. |  | Ref. |  | Ref. |  | Ref. |  | Ref. |
| Current smoker |  | **1.40 (1.25, 1.56)** |  | **1.76 (1.57, 1.97)** |  | **1.48 (1.28, 1.71)** |  | **1.14 (1.04, 1.26)** |  | **1.47 (1.32, 1.65)** |
| Ex-smoker |  | **1.39 (1.28, 1.52)** |  | **1.26 (1.14, 1.39)** |  | 1.09 (0.96, 1.23) |  | 1.07 (1.00, 1.16) |  | **1.18 (1.08, 1.30)** |
| LTPA |  |  |  |  |  |  |  |  |  |  |
| High |  | Ref. |  | Ref. |  | Ref. |  | Ref. |  | Ref. |
| Medium |  | 1.04 (0.93, 1.17) |  | 1.09 (0.97, 1.23) |  | **1.24 (1.05, 1.46)** |  | 1.02 (0.92, 1.12) |  | 1.05 (0.94, 1.18) |
| Low |  | **1.21 (1.08, 1.35)** |  | **1.25 (1.12, 1.41)** |  | **1.34 (1.14, 1.58)** |  | 1.09 (0.99, 1.20) |  | **1.14 (1.02, 1.28)** |
| None |  | **1.41 (1.24, 1.59)** |  | **1.39 (1.22, 1.58)** |  | **1.56 (1.31, 1.85)** |  | **1.16 (1.04, 1.29)** |  | **1.23 (1.09, 1.40)** |
| Diet quintile |  |  |  |  |  |  |  |  |  |  |
| 5 (healthiest) |  | Ref. |  | Ref. |  | Ref. |  | Ref. |  | Ref. |
| 4 |  | **1.20 (1.07, 1.35)** |  | **1.15 (1.01, 1.32)** |  | **1.22 (1.03, 1.44)** |  | 1.07 (0.97, 1.18) |  | 1.05 (0.91, 1.19) |
| 3 |  | **1.29 (1.13, 1.47)** |  | **1.20 (1.03, 1.38)** |  | 1.18 (0.98, 1.42) |  | 1.08 (0.97, 1.20) |  | **1.24 (1.08, 1.42)** |
| 2 |  | **1.31 (1.15, 1.49)** |  | **1.32 (1.14, 1.53)** |  | **1.32 (1.10, 1.57)** |  | 1.11 (1.00, 1.24) |  | **1.20 (1.05, 1.38)** |
| 1 |  | **1.30 (1.11, 1.52)** |  | **1.43 (1.22, 1.67)** |  | **1.38 (1.13, 1.70)** |  | **1.18 (1.04, 1.35)** |  | **1.36 (1.16, 1.59)** |
| Weekly alcohol consumption |  |  |  |  |  |  |  |  |  |  |
| 0 days |  | Ref. |  | Ref. |  | Ref. |  | Ref. |  | Ref. |
| 0-1 days |  | 0.90 (0.80, 1.01) |  | 0.98 (0.86, 1.11) |  | 0.96 (0.82, 1.12) |  | 0.94 (0.85, 1.05) |  | **0.87 (0.77, 0.99)** |
| >1-3 days |  | **0.82 (0.73, 0.93)** |  | **0.86 (0.75, 0.98)** |  | **0.64 (0.54, 0.76)** |  | **0.89 (0.80, 0.99)** |  | 0.92 (0.81, 1.05) |
| >3 days |  | **0.76 (0.67, 0.86)** |  | **0.82 (0.72, 0.94)** |  | **0.54 (0.45, 0.64)** |  | **0.88 (0.79, 0.98)** |  | **0.85 (0.74, 0.96)** |

*Note*: MetS, metabolic syndrome; Red., reduced; HDL, high-density lipoprotein; OR, odds ratio; CI, confidence interval; Ref., reference group; WC, white-collar; BC, blue-collar; LTPA, leisure-time physical activity.

^a^OR’s written in bold are significant (P<.05).
